# Cluster-Based Evaluation of Dietary Guideline Adherence and Food Literacy Among Adolescents: Implications for Tailored Diets

**DOI:** 10.3390/nu18020241

**Published:** 2026-01-12

**Authors:** Jimin Lim, Jieun Oh

**Affiliations:** 1 Department of Nutritional Science and Food Management, Ewha Womans University, Seoul 03760, Republic of Korea; jiminlim@ewha.ac.kr; 2 College of Science and Industry Convergence, Ewha Womans University, Seoul 03760, Republic of Korea

**Keywords:** dietary guideline adherence, food literacy, adolescent nutrition, cluster analysis, dietary behaviors

## Abstract

Background: Adolescence is a formative period for lifelong dietary patterns, yet Korean adolescents show low fruit and vegetable intake, high sugar and sodium consumption, and rising obesity, highlighting the importance of multidimensional assessment that integrates behavioral, cultural, environmental, and competency-related factors. Methods: A total of 1010 adolescents aged 12–18 years completed an online cross-sectional survey assessing food intake, dietary and physical activity behaviors, dietary culture, and Food Literacy (FL) competencies. Standardized scores were used for hierarchical and K-means clustering to identify dietary practice patterns, and between-cluster differences were examined using ANOVA. Correlation and regression analyses were conducted to examine associations between Dietary Guideline Adherence (DGA) and FL. Results: Four clusters were identified—selective intake–low support (20.4%), regular habits–unbalanced intake (33.3%), high adherence (23.2%), and low adherence (23.1%)—with significant differences in DGA total and domain scores (*p* < 0.001). The high-adherence cluster showed balanced intake, regular routines, and strong household support, whereas the low-adherence cluster showed poor diet quality, irregular behaviors, and lower socioeconomic status. FL differed across clusters (*p* < 0.001) and correlated with DGA (r = 0.496, *p* < 0.01). Total FL predicted DGA (β = 0.496, *p* < 0.001), explaining 25% of its variance (R^2^ = 0.246). Conclusions: Adolescent diet quality appears to be associated with behavioral, cultural, and competency-related factors. These findings suggest that cluster-specific strategies—such as fat–sugar–sodium reduction, promotion of low-sodium and diverse diets, and maintenance of balanced-dietary patterns—may support tailored school- and community-based nutrition programs and inform further longitudinal and intervention research.

## 1. Introduction

Adolescence represents a transitional stage between childhood and adulthood, characterized by rapid physical growth as well as cognitive and emotional development. It is also a critical period during which lifelong health-related behaviors are established. As adolescents gain greater independence, their lifestyle and dietary habits are increasingly shaped within the contexts of family, school, community, and cultural influences. Establishing healthy eating patterns and engaging in adequate physical activity during this stage plays a crucial role in reducing the risk of obesity and chronic diseases in adulthood, thereby enhancing long-term health and quality of life [1,2,3].

In response to these concerns, many countries have developed dietary guidelines aimed at promoting healthy eating behaviors across populations, including adolescents. The Dietary Guidelines for Americans are periodically revised to improve nutritional status and overall health [4,5,6], highlighting inadequate fruit and vegetable consumption as well as excessive intake of sugar-sweetened beverages and sodium among adolescents as major issues [7,8]. Similarly, the Dietary Guidelines for Koreans (DGK) emphasize the importance of balanced food intake, regular physical activity, and the development of sustainable dietary cultures [9]. However, findings from the 2016–2019 Korea National Health and Nutrition Examination Survey (KNHANES) indicated that only 1.4% of adolescents met the recommended intake levels for both fruits and vegetables, whereas 79.5% failed to meet the recommendations for either food group [10]. In contrast, sugar-sweetened beverage intake remained high; adolescents aged 12–18 years consumed an average of 46–55 g of free sugars per day, accounting for more than 10% of their total energy intake [11]. Sodium intake also substantially exceeded the WHO recommended limits, with the rising consumption of processed and fast foods has been further identified as a key risk factor contributing to the deterioration of diet quality in this age group [12].

Consistent with these dietary patterns, the prevalence of obesity among Korean adolescents more than doubled, from 5.6% in 2011 to 13.4% in 2021, with one of the steepest increases reported among OECD countries [13]. Such trends are far from aligned with the goals of promoting healthy and sustainable diets [14]. Moreover, the affordability and wide availability of ultra-processed foods reinforce and entrench unhealthy eating behaviors among adolescents [15]. Therefore, guiding adolescents toward healthier and more sustainable dietary practices requires not only individual-level efforts but also systemic changes within the broader food environment.

While the family remains a key anchor for adolescents [16], their growing independence in food choice has also been linked to negative perceptions of parental influence. Evidence indicates a rising prevalence of meals consumed outside the home, accompanied by greater intake of energy-dense, processed, and “trendy” foods, which have been linked to less healthy dietary patterns [17,18]. Nevertheless, parental education and the household food environment continue to exert strong influence, with evidence that parental role modeling and socioeconomic factors play a decisive role in shaping adolescent eating behaviors [19,20]. These dynamics suggest that adolescent dietary practices cannot be fully explained by nutrient intake indicators alone; rather, they require a multidimensional approach that considers food choices, dietary behaviors (e.g., meal regularity and physical activity), and cultural or environmental factors (e.g., family support and individual practices).

To address this complexity, the Dietary Guideline Adherence (DGA) tool—developed based on the DGK—has been employed as a framework for comprehensively evaluating adolescents’ dietary intake, dietary and physical activity behaviors, and dietary culture [21]. At the same time, increasing attention has been directed toward understanding dietary practices through the lens of practical competencies in everyday life. Food literacy (FL)—defined as the knowledge, skills, attitudes, and behaviors required for planning, selecting, preparing, and consuming food [22]—is a critical capacity developed during adolescence [23,24] and has a lasting influence on the establishment of healthy and sustainable dietary habits in adulthood [25,26].

Accordingly, this study aimed to (1) apply the DGA tool to comprehensively assess adolescents’ dietary intake, dietary behaviors and physical activity, and dietary culture in order to identify clusters and their sociodemographic correlates, and (2) incorporate FL as a complementary indicator to examine whether these clusters align with adolescents’ practical dietary competencies.

## 2. Materials and Methods

### 2.1. Settings and Participants

A cross-sectional online survey was conducted between May and August 2023. Participants were Korean adolescents aged 12–18 years who completed a web-based self-administered questionnaire after being informed of the study’s aims and procedures and providing consent. A stratified sampling design was employed, with 25% of respondents recruited from county-level rural areas. To ensure representativeness, the sample size was calculated to achieve a margin of error less than 0.04 at the 95% confidence level. In total, 1010 adolescents from 17 provinces, which were reclassified into six regions for analytical purposes (the Capital, Gangwon, Chungcheong, Jeolla, Gyeongsang, and Jeju) were included in the final analysis after excluding incomplete or duplicate responses. Ethical approval was obtained from the Institutional Review Board of Sangmyung University (IRB-SMU-C-2023-1-0015).

### 2.2. Survey Instrument

Dietary behaviors were assessed using the DGA tool, developed in line with the DGK [21]. The instrument consists of 24 items across three domains: food intake, dietary and physical activity behaviors, and dietary culture. Items were rated on a five-point Likert scale, with some reverse-coded, and higher scores indicated greater adherence. FL was measured using a validated 19-item tool that covers five domains: selection, production, distribution, preparation/cooking, and intake [27]. Higher scores represented stronger competencies in healthy food practices and sustainability awareness. Sociodemographic variables included sex, age, grade, residential area, household type, and economic status. Self-reported height and weight were collected to calculate body mass index (BMI).

### 2.3. Data Analysis

**Figure A1:**
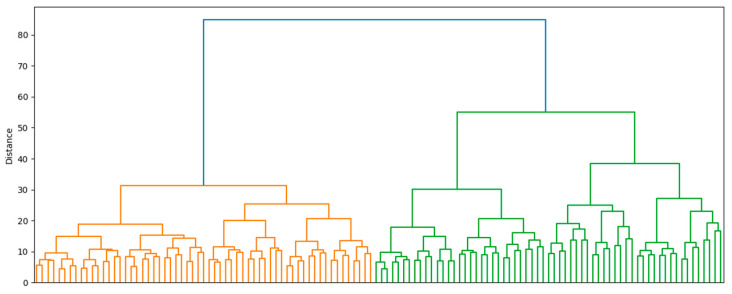
Dendrogram of hierarchical clustering using Ward’s method based on standardized variables. The colored branches in the dendrogram are provided for visual distinction of hierarchical groupings and do not represent the final cluster solution. The analysis ultimately retained four clusters.

To identify patterns of dietary practices among adolescents, cluster analysis was conducted. This method classifies individuals into mutually exclusive groups based on similarity, such that participants within a cluster share similar characteristics, while differences are maximized between clusters [28,29]. A two-step strategy was employed to determine the number of clusters. First, hierarchical cluster analysis using Ward’s method [30] with squared Euclidean distance was applied to explore potential clustering solutions [31]. Examination of the scree plot and dendrogram suggested a four-cluster solution as a meaningful and interpretable structure (Appendix A Figure A1). Subsequently, K-means cluster analysis was performed to finalize group allocation, with different initial seeds tested to ensure stability.

Clustering was performed using standardized (z-score) values of the 24 individual items from the DGA assessment, encompassing food intake, dietary and physical activity behaviors, and dietary culture domains. Although the DGA tool provides weighted scores (0–100) reflecting domain importance, all items were standardized (z-scores, mean = 0, SD = 1) prior to clustering so that each variable contributed equally [28,32]. The four derived clusters were retained without reclassification [33,34,35].

**Figure A2:**
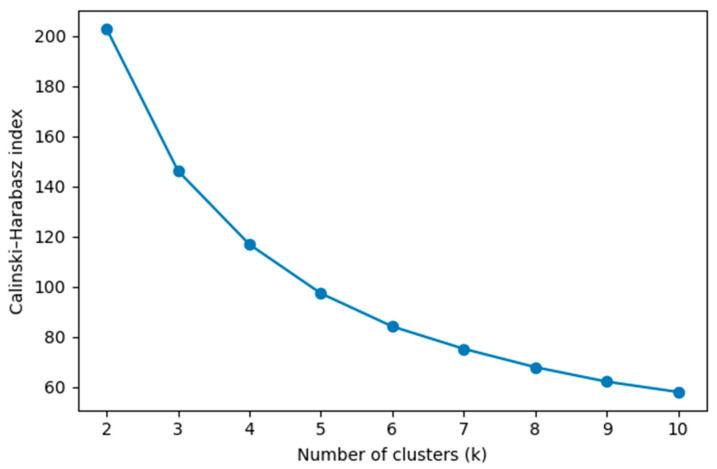
Calinski–Harabasz index values for k-means clustering solutions with different numbers of clusters (k = 2–10).

As a reference measure, the Calinski–Harabasz index was calculated for clustering solutions with varying numbers of clusters (k = 2–10) to evaluate relative cluster separation (Appendix A Figure A2) [36]. Although the index was highest for solutions with fewer clusters, the four-cluster solution was retained based on a balance between statistical separation and interpretability, consistent with prior clustering studies in nutrition and public health research [37,38].

Between cluster differences were examined by comparing mean scores across clusters using one-way analysis of variance (ANOVA) with Tukey’s post hoc tests. Chi-square tests were conducted to examine differences in the distribution of categorical variables [39]. To explore associations between DGA and FL, Pearson’s correlation analysis was first used to examine domain-level association patterns, and simple linear regression analysis was subsequently conducted using composite scores to quantify the overall association and explained variance. Normality of the variables was assessed using the Shapiro–Wilk test. Descriptive statistics were used to summarize sociodemographic characteristics. All analyses were conducted using SPSS version 29.0.2 (IBM Corp., Armonk, NY, USA), and statistical significance was set at *p* < 0.05. In addition, cluster validity indices, dendrograms, and graphical visualizations were generated using Python version 3.14 (Python Software Foundation, Wilmington, DE, USA) with pandas, NumPy, scikit-learn, SciPy, and matplotlib.

## 3. Results

### 3.1. Cluster Profiles and Characteristics

**Figure 1:**
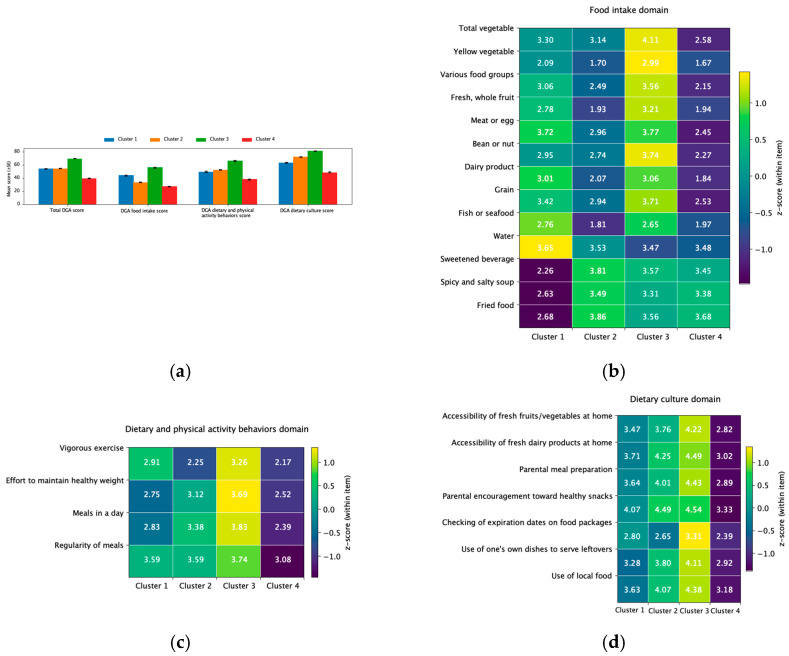
Visualized differences in total, domain-level, and item-level DGA scores across clusters: (**a**) Total and domain-level DGA scores; (**b**) Food intake domain items; (**c**) Dietary and physical activity behaviors domain items; (**d**) Dietary culture domain items.

A four-cluster solution was identified through the two-step cluster analysis (cluster 1: n = 206, 20.4%; cluster 2: n = 336, 33.3%; cluster 3: n = 235, 23.2%; cluster 4: n = 233, 23.1%). As shown in Figure 1a, total DGA scores and all three domain scores (food intake, dietary and physical activity behaviors, and dietary culture) differed significantly across clusters (*p* < 0.001). Cluster 3 yielded the highest overall adherence (total score = 69.61; food intake = 55.75; dietary and physical activity behaviors = 66.06; dietary culture = 80.71), whereas cluster 4 showed the lowest scores across all domains (total = 39.54; food intake = 26.83; dietary and physical activity behaviors = 37.81; dietary culture = 48.59). Cluster 1 demonstrated relatively high food intake (43.70) but below-average scores in dietary and physical activity behaviors (49.56) and dietary culture (62.93), while cluster 2 showed the opposite pattern, with low food intake scores (33.46) but higher scores in dietary and physical activity behaviors (52.39) and dietary culture (72.05).

Significant differences were also observed at the item level (Table 1, Figure 1b–d). In the food intake domain, cluster 3 reported the highest consumption of vegetables (total and yellow), fresh fruits, beans/nuts, and grains, while cluster 4 showed the lowest intakes (all *p* < 0.001). Cluster 1 had the lowest scores on reverse-coded items, including sweetened beverages, salty soups, and fried foods, indicating the highest consumption of these items. In contrast, cluster 2 scored highest on these items, indicating the lowest intake (*p* < 0.001). In the dietary and physical activity behaviors domain, cluster 3 reported significantly higher levels of vigorous exercise, weight management, daily meal frequency, and meal regularity. Meanwhile, cluster 4 scored lowest on meal frequency and regularity (*p* < 0.001). In the dietary culture domain, cluster 3 demonstrated the strongest household support (e.g., accessibility of fruits/vegetables and dairy products, as well as parental meal preparation) and personal practices (e.g., checking expiration dates, using one’s own dishes, and using local food). Meanwhile, cluster 4 consistently scored lowest across all items (*p* < 0.001).

Based on these patterns, the clusters were labeled as follows: selective intake–low support pattern (cluster 1), regular habits–unbalanced intake pattern (cluster 2), high adherence pattern (cluster 3), and low adherence pattern (cluster 4), in line with previous studies that named clusters according to dietary intake and lifestyle characteristics [33,40,41].

### 3.2. Selection of Items and Final Scales for Each Dietary Guideline Domain

Significant differences among clusters were observed for BMI (*p* = 0.017), sex (*p* < 0.001), residential regions (*p* = 0.019), and economic status (*p* < 0.001), and no differences were found for age, weight status, grade, or household type (Table 2). Cluster 1 showed the highest mean BMI (22.31 kg/m^2^), while cluster 2 had the lowest (21.21 kg/m^2^). In terms of sex distribution, cluster 1 had a higher proportion of boys (66%), whereas cluster 2 (62.5%), cluster 3 (50.6) and cluster 4 (59.7%) had a higher proportion of girls. The distribution of clusters differed significantly across the six regions (χ^2^ = 28.441, *p* < 0.05), indicating that cluster membership was associated with regional classification. With respect to economic status, clusters 1 and 3 included more adolescents from high or upper-middle households, whereas cluster 4 included more from low or lower-middle households.

### 3.3. Distribution of Component Grades Across Clusters

Analysis of total DGA and domain-specific grades showed that approximately 25% of participants were classified as “Excellent,” 50% as “Fair,” and 25% as “Poor” (Table 3).

In the food intake domain, cluster 3 had the highest proportion of “Excellent” grades (74.5%) and no cases in the “Poor” category, indicating the strongest dietary intake. By contrast, cluster 4 showed the highest proportion of “Poor” grades (57.9%), while clusters 1 and 2 were predominantly “Fair” (59.2% and 68.5%, respectively; *p* < 0.001). In the dietary and physical activity behaviors domain, cluster 3 again scored highest (“Excellent” = 58.7%, “Poor” = 3%), whereas cluster 4 showed the lowest performance with 58.4% in the “Poor” category. Clusters 1 and 2 were mostly “Fair” (59.2% and 61.0%; *p* < 0.001). In the dietary culture domain, cluster 3 showed the strongest adherence (“Excellent” = 60.0%, “Poor” = 0%), while cluster 4 showed the weakest adherence, with 77.3% rated “Poor.” Clusters 1 and 2 were largely “Fair” (61.7% and 68.2%; *p* < 0.001). Overall, cluster 3 consistently demonstrated high adherence across all domains, cluster 4 showed low adherence, and clusters 1 and 2 reflected intermediate levels of practice.

### 3.4. Food Literacy Scores and Grades Across Clusters

Significant differences were observed in total FL scores and across all five domains—selection, production, distribution, preparation/cooking, and intake (*p* < 0.001; Table 4).

Cluster 3 demonstrated the highest overall FL score (64.06 ± 15.68) and consistently outperformed the other clusters in every domain (selection 72.47 ± 16.36, production 48.30 ± 26.57, distribution 61.71 ± 24.69, preparation/cooking 71.34 ± 19.15, intake 66.01 ± 18.62). In contrast, cluster 4 had the lowest total score (44.05 ± 15.02) and scored lowest in each domain: selection (51.03 ± 18.30), production (29.12 ± 20.73), distribution (38.40 ± 23.15), preparation/cooking (57.16 ± 22.36), and intake (45.74 ± 21.44). Cluster 1 (52.72 ± 14.56) and cluster 2 (50.96 ± 14.39) fell within the medium range, with cluster 1 scoring particularly low in selection and production, and cluster 2 scoring below average in all domains except selection.

Grade distributions also differed significantly across clusters (*p* < 0.001). Overall, 28.0% of adolescents were classified as “Upper,” 28.7% as “Upper middle,” 21.6% as “Lower middle,” and 21.7% as “Lower.” Cluster 3 had the highest proportion in the “Upper” category (56.6%) and the lowest proportion in the “Lower” category (6.8%). In contrast, cluster 4 had the smallest proportion in the “Upper” group (9.9%) and the largest proportion in the “Lower” group (38.2%). Clusters 1 and 2 were mainly distributed in the “Upper middle” category (31.6% and 33.9%, respectively).

### 3.5. Association Between Food Literacy and Dietary Guideline Adherence

As shown in Table 5, total DGA scores were positively correlated with total FL scores (r = 0.496, *p* < 0.01). At the domain level, consistent positive correlation patterns were observed between DGA and FL, providing insight into how specific components of food literacy relate to dietary guideline adherence. In particular, relatively stronger associations were observed for FL selection (r = 0.437) and intake (r = 0.429). Similarly, positive correlations were observed between total FL and DGA domains, including food intake (r = 0.407), dietary and physical activity behaviors (r = 0.334), and dietary culture (r = 0.402).

Building on these domain-level association patterns, regression analysis was conducted to quantify the overall relationship at the composite-score level. As shown in Table 6, total FL was a significant predictor of total DGA (β = 0.50, t = 18.13, *p* < 0.001), explaining approximately 25% of the variance (R^2^ = 0.25, F = 328.70, *p* < 0.001). Specifically, a one-point increase in FL score was associated with a 0.67-point increase in DGA score (B = 0.672).

## 4. Discussion

This study identified four clusters of Korean adolescents by comprehensively evaluating food intake, dietary and physical activity behaviors, and dietary culture based on the DGK. Even with comparable total DGA scores, each cluster exhibited distinct strengths and weaknesses across subdomains, underscoring that adolescent dietary behaviors cannot be sufficiently explained by a single intake indicator alone. Furthermore, by incorporating FL into the analysis, this study clarified how multidimensional dietary competencies—including food selection, production, distribution, preparation and cooking, and intake—are associated with actual dietary quality.

The relationship between FL and DGA was also evident: the two indicators showed a significant quantitative correlation, with the FL total score explaining approximately 25% of the variance in the DGA total score. Specifically, a one-point increase in FL corresponded to a 0.67-point increase in DGA, suggesting that adherence to dietary guidelines among adolescents is closely tied not only to knowledge but also to practical competencies in food selection, cooking, and intake. This finding is consistent with previous research reporting a strong concordance between DGA and the Nutrition Quotient (NQ) [21] as well as significant associations between the NQ and FL, thereby strengthening the validity of the present interpretation [42,43]. Consequently, the cluster-specific characteristics observed in this study can be understood within the broader context of competencies that extend beyond food intake alone.

At the same time, the explanatory power of FL should be interpreted with appropriate caution. Although FL accounted for a statistically significant proportion of variance in dietary guideline adherence, approximately 75% of the variance remained unexplained, indicating that adolescent dietary behaviors are inherently multifactorial. Factors beyond individual competencies—such as food environment, household economic access, school and community contexts, family support, and mental health—are likely to play a substantial role in shaping dietary practices.

In addition, all variables in this study were assessed using self-reported data, which may be subject to recall bias and social desirability bias. The absence of objective measures of dietary intake or physical activity limits external validation of the identified patterns and raises the possibility that the observed clusters may partially reflect differences in response tendencies rather than true behavioral differences alone. Therefore, FL should be regarded as one important determinant among multiple interacting factors influencing dietary guideline adherence, rather than as a sole or dominant predictor. Future studies incorporating objective dietary assessments and broader contextual variables are needed to further validate and refine these findings.

Previous studies on adolescent diets, both domestic and international, have primarily relied on intake indicators such as food frequency or nutrient intake to evaluate dietary quality [28,44,45,46] and its associations with demographic factors such as sex, age, household income, and parental education. While valuable, such approaches often fail to capture the contextual and behavioral dimensions underlying adolescents’ actual dietary practices [33,47,48,49]. By contrast, the present study addressed these limitations by adopting a multidimensional framework that integrated food intake, behavioral elements such as meal regularity and physical activity, and cultural factors such as household support and personal practices. This approach revealed that groups with similar DGA scores nevertheless differed in subdomains and FL levels [50,51], highlighting the importance of multidimensional indicators for assessing adolescent dietary behaviors.

### 4.1. Evaluation of Checklist Alignment with Korean Dietary Guidelines

Cluster 1 (selective intake–low support pattern) exhibited high intake scores across various food groups, suggesting balanced consumption. However, it also showed a high intake of foods that require restraint, such as sweetened beverages, fried foods, and salty soups, resulting in overall poor diet quality. Additionally, in terms of dietary behavior, meal frequency was low. In the food culture domain, it exhibited characteristics of insufficient social support, such as inadequate meal preparation at home and low parental support. This aligns with prior research indicating that adolescents often find it challenging to maintain healthy eating habits when parental support is low [52] and exhibit more desirable dietary patterns when experiencing structured food parenting that respects autonomy [53,54]. Demographically, the cluster had a relatively higher proportion of male students and the highest average BMI among the clusters. This aligns with prior research showing male students consume more energy-dense snacks, sweetened beverages, and fast food than female students [55,56,57] and also have higher rates of skipping breakfast than their female counterparts [58]. Skipping breakfast is closely associated with increased consumption of high-fat, high-sugar, and high-sodium foods [59,60], as well as higher BMI, which may potentially increase the risk of weight gain and obesity in the long term [61,62,63]. Despite moderate overall FL, this cluster showed particular weaknesses in the selection and production domains, including limited skills in evaluating quality, interpreting food information, and considering sustainability. This aligns with evidence that boys often have lower FL than girls in hygiene, cultural, and environmental aspects [64,65]. Therefore, interventions should prioritize a “fat–sugar–sodium reduction” strategy, complemented by healthier cooking methods, beverage choices, and family-based support such as cooking and preparing meals together. Enhancing adolescents’ own competencies in food choice and production through structured education is also crucial [66], given their growing autonomy in food purchasing and dining-out practices [67].

Cluster 2 (regular habits–unbalanced intake pattern) was characterized by a higher proportion of girls and exhibited stability in dietary behaviors and culture, including regular meals, strong parental support, and the lowest intake of sugar-sweetened beverages and fried foods among the clusters. However, this group demonstrated marked imbalance in food choices, with insufficient consumption of vegetables, fruits, and fish/seafood. While its overall FL score was moderate, only the selection domain exceeded the mean, suggesting an awareness of healthy selection that was not translated into actual intake [68]. This discrepancy indicates that environmental and social support alone may be insufficient if adolescents lack individual competencies in food preparation and consumption, consistent with evidence that mere food availability does not guarantee intake unless accessibility—such as ready-to-eat or prepared foods—is ensured [69,70,71]. These gaps may partly relate to the relatively higher proportion of girls in this cluster, as prior studies have shown that female adolescents are more prone than male adolescents to restrictive eating for weight control [72,73] as well as to emotional eating [74,75,76], both of which can reduce healthy food group consumption [77,78]. However, such patterns likely reflect broader structural factors common to adolescents, including time constraints, reliance on school meals, and limited exposure to a diverse range of foods [79]. Accordingly, this cluster may be defined as possessing stable lifestyle habits but insufficient diversity in food intake due to limited preparation and intake skills. Intervention strategies should therefore emphasize a “low-sodium and diverse diet” approach, maintaining control of discretionary foods while prioritizing an increased intake of vegetables, fruits, and fish/seafood. Practical approaches include offering fresh produce as snacks, incorporating fish and seafood as protein sources, and adjusting school and household menus to ensure exposure to a diverse range of food groups. Community-level nutrition education and programs targeting emotional eating could further strengthen food diversity, particularly when coordinated across schools, families, and local organizations [80].

Cluster 3 (high adherence pattern) demonstrated the highest scores across food intake, dietary behaviors, and dietary culture, as well as the highest FL across all five domains when compared to the other clusters. This group represented the healthiest pattern, suggesting that strong adherence to dietary guidelines is closely aligned with multidimensional competencies in food selection, preparation, intake, and production [81]. The relatively higher proportion of adolescents from middle to upper socioeconomic backgrounds also reflects previous evidence that greater household resources and parental support facilitate better diet quality [82,83,84]. Although no specific vulnerabilities were identified, adolescence is a period of increasing autonomy and susceptibility to peer and media influences [85,86,87]. Maintaining these positive practices, therefore, requires sustained environmental support, as dietary habits are dynamic and shaped by broader contexts over time [88,89]. For this group, a “balanced diet” strategy is most appropriate. Maintaining the current diversity of food intake is essential. At the same time, school and community initiatives could help reinforce positive habits by promoting exemplary dietary practices and encouraging peer-to-peer sharing of healthy eating experiences. Such reinforcement may support the long-term continuation of healthy dietary behaviors established during adolescence.

Cluster 4 (low adherence pattern) was the most vulnerable group, characterized by lower socioeconomic status and the lowest scores across food intake, dietary behaviors, dietary culture, and all FL domains. This cluster demonstrated an insufficient intake of fruits, vegetables, and protein sources, coupled with a high reliance on energy-dense foods rich in fat, sugar, and sodium. Its members reported irregular and infrequent meals, low levels of physical activity, and minimal parental support in terms of meal preparation and encouragement. Weaknesses across all FL domains—particularly in selection, production, and distribution—reflect not only poor intake but also structural limitations in food management, preparation, and intake. These vulnerabilities appear closely linked to socioeconomic constraints, as limited-income households prioritize affordability over nutritional quality [90,91,92] often perceiving healthy foods as more costly [93] and time-consuming, which further increases reliance on convenience foods [94,95]. Such constraints also restrict opportunities for physical activity [96,97] and reduce parental involvement [98] in dietary support. Targeted interventions should prioritize a “low-sodium and diverse diet” strategy, focusing on expanding access to vegetables and fruits, introducing low-sodium options at schools, and encouraging shared family meals while also reducing dependence on high-sodium convenience foods [99,100]. Enhancing dietary diversity is essential, and should be combined with initiatives to increase physical activity, such as strengthening school-based physical education, expanding access to sports programs, and encouraging outdoor family activities. These combined approaches may help address the multifaceted vulnerabilities of this group.

### 4.2. Implications

This study demonstrated that adolescent dietary patterns can be classified multidimensionally by integrating food intake, behaviors, and culture through the DGA indicator, and further contextualized by incorporating FL. This approach highlights the importance of examining interactions among domains and competencies, rather than relying solely on intake-based indicators. Notably, the strong associations observed between the selection and intake domains of FL and overall DGA scores suggest that adolescents’ practical abilities to select and consume healthy foods represent an important component of dietary guideline adherence among multiple interacting factors, supporting the validity of the DGA framework while underscoring the need for a comprehensive perspective.

From a policy perspective, school- and community-based programs should be tailored to cluster-specific differences, combining strategies such as discretionary food control, dietary diversity, regular meal promotion, and household support. For instance, in groups where a gap between awareness and practice is evident (e.g., cluster 2), interventions that strengthen preparation and cooking competencies and improve access to affordable and nutritious foods may foster meaningful behavioral changes. Such strategies may include increasing the availability of minimally processed foods, promoting simplified cooking methods, and diversifying school menus, all of which can be effective in fostering these changes. For socioeconomically disadvantaged groups (e.g., cluster 4), providing low-cost balanced diets, subsidizing access to fruits and vegetables, and enhancing community-level availability of fresh foods are essential.

Academically, conducting a combined analysis of FL and DGA demonstrates the potential to complement and enrich the interpretation of adolescent dietary assessments and suggests opportunities for developing new tools that combine these indicators. Practically, dietitians, parents, and community stakeholders could utilize checklists or educational resources tailored to cluster-specific needs, enabling more concrete and actionable guidance. Ultimately, creating a multilayered environment that links education, school meals, households, and communities is critical to ensuring that adolescents’ healthy food choices are consistently translated into actual dietary practices.

### 4.3. Limitations

This study has several limitations. First, its cross-sectional design prevents causal inferences between DGA, FL, and health outcomes. Second, both indices were derived from self-reported questionnaires, which may be subject to recall and social desirability biases. In addition, dietary intake patterns and physical activity levels were not assessed using objective measures, such as dietary records or device-based physical activity assessments, limiting external validation of the identified clusters. As a result, the observed clustering patterns may partially reflect differences in response tendencies rather than true behavioral differences alone. Third, body mass index (BMI) was calculated based on self-reported height and weight. Previous research has shown that adolescents tend to underreport body weight, which may lead to systematic underestimation of BMI and obesity prevalence. Accordingly, the observed associations between cluster membership and obesity-related outcomes should be interpreted with caution, as these relationships may be under- or overestimated due to reporting bias. Fourth, as the DGA and FL tools were developed based on Korean guidelines and contexts, their generalizability to adolescents in other cultural settings is limited. Fourth, psychosocial factors such as peer influence, media exposure, community environment, and emotional eating were not fully captured by the survey-based measures. Finally, the clustering results may be influenced by methodological choices, including the use of Ward’s hierarchical clustering with squared Euclidean distance. Although this approach was selected to maximize interpretability and cluster homogeneity, alternative distance metrics or linkage methods may yield different clustering structures. In addition, the cluster-specific intervention strategies proposed herein remain theoretical and require empirical validation in real-world settings.

Future research should employ longitudinal designs to examine how adolescent DGA and FL levels predict adult dietary behaviors and health outcomes over time, incorporate objective dietary and physical activity measures to validate self-reported patterns, conduct cross-cultural comparative studies, and evaluate the effectiveness of tailored interventions through experimental or implementation-based approaches.

## 5. Conclusions

This study evaluated DKG adherence among Korean adolescents using the DGA across multiple domains and identified four distinct clusters with varying strengths and vulnerabilities. Even with similar total scores, differences in subdomains and FL levels highlighted the inadequacy of single indicators for explaining adolescent dietary behaviors. Moreover, significant associations between FL and DGA, with FL predicting adherence levels, underscore the close linkage between adolescents’ competencies and their guideline compliance.

These findings provide academic evidence for adopting multidimensional approaches that integrate intake, behaviors, culture, and competencies in adolescent dietary assessment. At the policy level, the proposed tailored strategies (e.g., fat–sugar–sodium reduction, low-sodium and diverse diet, balanced diet) offer a practical basis for designing school- and community-based nutrition programs. Practically, they may guide actionable interventions in households and food service settings. Future studies should adopt longitudinal designs to clarify how adolescent dietary characteristics shape adult health trajectories and endeavor to develop culturally sensitive, context-specific intervention strategies to enhance sustainable dietary practices.

## Figures and Tables

**Table 1:** Weighted mean (SE) of total and domain-level Dietary Guideline Adherence (DGA) scores by cluster ^1^ among adolescents aged 12 to 18 years.

	Total (N = 1010)	Cluster 1 (N = 206, 20.4%)	Cluster 2 (N = 336, 33.3%)	Cluster 3 (N = 235, 23.2%)	Cluster 4 (N = 233, 23.1%)	F ^2^
	Weighted mean (weighted SE)					
**Total DGA score (overall)**	54.46 (0.38)	54.07 (0.49) ^b3^	54.44 (0.31) ^b^	69.61 (0.45) ^c^	39.54 (0.43) ^a^	846.77 ***
**D** **omain-level (subtotal) scores**						
DGA food intake	39.21 (0.45)	43.70 (0.76) ^c^	33.46 (0.45) ^b^	55.75 (0.70) ^d^	26.83 (0.60) ^a^	415.4 ***
DGA dietary and physical activity behaviors	51.63 (0.52)	49.56 (0.97) ^b^	52.39 (0.72) ^b^	66.06 (0.95) ^c^	37.81 (0.84) ^a^	171.22 ***
DGA dietary culture	66.79 (0.48)	62.93 (0.78) ^b^	72.05 (0.48) ^c^	80.71 (0.61) ^d^	48.59 (0.76) ^a^	439.93 ***
**Food intake item-level scores**						
Total vegetables	3.27 (0.03)	3.30 (0.06) ^b^	3.14 (0.05) ^b^	4.11 (0.05) ^c^	2.58 (0.06) ^a^	133.89 ***
Yellow vegetables	2.07 (0.03)	2.09 (0.07) ^b^	1.70 (0.05) ^a^	2.99 (0.08) ^c^	1.67 (0.05) ^a^	103.13 ***
Various food groups	2.78 (0.04)	3.06 (0.08) ^c^	2.49 (0.05) ^b^	3.56 (0.08) ^d^	2.15 (0.06) ^a^	84.88 ***
Fresh, whole fruits	2.40 (0.04)	2.78 (0.08) ^b^	1.93 (0.05) ^a^	3.21 (0.08) ^c^	1.94 (0.06) ^a^	99.34 ***
Meat or eggs	3.19 (0.04)	3.72 (0.07) ^c^	2.96 (0.06) ^b^	3.77 (0.07) ^c^	2.45 (0.07) ^a^	87.9 ***
Beans or nuts	2.91 (0.04)	2.95 (0.08) ^b^	2.74 (0.06) ^b^	3.74 (0.07) ^c^	2.27 (0.06) ^a^	78.17 ***
Dairy products	2.44 (0.04)	3.01 (0.08) ^b^	2.07 (0.06) ^a^	3.06 (0.08) ^b^	1.84 (0.06) ^a^	82.42 ***
Grains	3.12 (0.04)	3.42 (0.08) ^c^	2.94 (0.07) ^b^	3.71 (0.07) ^d^	2.53 (0.08) ^a^	43.03 ***
Fish or seafood	2.23 (0.03)	2.76 (0.07) ^b^	1.81 (0.04) ^a^	2.65 (0.06) ^b^	1.97 (0.06) ^a^	72.08 ***
Water	3.53 (0.07)	3.65 (0.15)	3.53 (0.11)	3.47 (0.14)	3.48 (0.13)	0.3
Sweetened beverages	3.35 (0.04)	2.26 (0.07) ^a^	3.81 (0.05) ^c^	3.57 (0.06) ^b^	3.45 (0.07) ^b^	117.68 ***
Spicy and salty soup	3.25 (0.03)	2.63 (0.06) ^a^	3.49 (0.04) ^b^	3.31 (0.06) ^b^	3.38 (0.06) ^b^	48.09 ***
Fried foods	3.51 (0.03)	2.68 (0.06) ^a^	3.86 (0.04) ^c^	3.56 (0.06) ^b^	3.68 (0.06) ^bc^	90.74 ***
**Dietary and physical activity behaviors item-level scores**						
Vigorous exercise	2.60 (0.04)	2.91 (0.08) ^b^	2.25 (0.05) ^a^	3.26 (0.08) ^c^	2.17 (0.06) ^a^	61.31 ***
Effort to maintain healthy weight	3.04 (0.04)	2.75 (0.08) ^a^	3.12 (0.06) ^b^	3.69 (0.07) ^c^	2.52 (0.06) ^a^	49.83 ***
Meals in a day	3.14 (0.03)	2.83 (0.07) ^b^	3.38 (0.05) ^c^	3.83 (0.06) ^d^	2.39 (0.06) ^a^	106.24 ***
Regularity of meals	3.50 (0.07)	3.59 (0.04) ^b^	3.59 (0.03) ^b^	3.74 (0.04) ^c^	3.08 (0.05) ^a^	52.14 ***
**Dietary culture item-level scores**						
Accessibility of fresh fruits/vegetables at home	3.59 (0.03)	3.47 (0.07) ^b^	3.76 (0.05) ^c^	4.22 (0.06) ^d^	2.82 (0.07) ^a^	88.59 ***
Accessibility of fresh dairy products at home	3.91 (0.03)	3.71 (0.06) ^b^	4.25 (0.04) ^c^	4.49 (0.05) ^d^	3.02 (0.06) ^a^	149.03 ***
Parental meal preparation	3.78 (0.03)	3.64 (0.06) ^b^	4.01 (0.04) ^c^	4.43 (0.04) ^d^	2.89 (0.05) ^a^	184.09 ***
Parental encouragement toward healthy snacking	4.15 (0.03)	4.07 (0.07) ^b^	4.49 (0.04) ^c^	4.54 (0.05) ^c^	3.33 (0.08) ^a^	100.63 ***
Checking of expiration dates on food packaging	2.78 (0.03)	2.80 (0.06) ^b^	2.65 (0.04) ^b^	3.31 (0.06) ^c^	2.39 (0.06) ^a^	46.94 ***
Use of one’s own dishes to serve leftovers	3.56 (0.03)	3.28 (0.07) ^b^	3.80 (0.05) ^c^	4.11 (0.06) ^d^	2.92 (0.07) ^a^	75.63 ***
Use of local food	3.85 (0.03)	3.63 (0.07) ^b^	4.07 (0.05) ^c^	4.38 (0.05) ^d^	3.18 (0.08) ^a^	72.47 ***

^1^ The four clusters have been identified using a cluster analysis of the DGA scores, and they have been assigned descriptive names that characterize the dietary adherence patterns of each cluster: Cluster 1 (selective intake-low support), Cluster 2 (regular habits-unbalanced intake), Cluster 3 (high adherence), Cluster 4 (low adherence); ^2^ *p*-values are from F tests on cluster differences (*** *p* < 0.001); ^3^ Values within a row with identical letters were significantly different, as determined by analysis of variance with Tukey post hoc tests.

**Table 2:** Weighted mean and sample size of demographics by cluster ^1^ for adolescents aged 12 to 18 years.

Demographics	Total (N = 1010)	Cluster 1 (N = 206, 20.4%)	Cluster 2 (N = 336, 33.3%)	Cluster 3 (N = 235, 23.2%)	Cluster 4 (N = 233, 23.1%)	F/χ^2 2^
	Weighted mean (weighted SE)					
Age (years)	15.98 (0.05)	15.83 (0.12)	16.11 (0.09)	15.91 (0.12)	15.99 (0.11)	1.32
BMI (kg/m^2^) ^3^	21.68 (0.14)	22.31 (0.33) ^b5^	21.21 (0.21) ^a^	21.44 (0.29) ^ab^	22.03 (0.32) ^ab^	3.41 *
**Weight status** ^4^	Sample size (weighted %)					
Underweight	213 (21.1)	36 (17.5)	74 (22)	53 (22.6)	50 (21.5)	9.53
Normal	515 (51)	100 (48.5)	180 (53.6)	124 (52.8)	111 (47.6)	
Overweight	119 (11.8)	27 (13.1)	36 (10.7)	26 (11.1)	30 (12.9)	
Obesity	163 (16.1)	43 (20.9)	46 (13.7)	32 (13.6)	42 (18)	
**Sex**						
Boy	472 (46.7)	136 (66)	126 (37.5)	116 (49.4)	94 (40.3)	46.76 ***
Girl	538 (53.3)	70 (34)	210 (62.5)	119 (50.6)	139 (59.7)	
**Grade**						
Middle school (12~14 years old)	287 (28.4)	63 (30.6)	84 (25)	72 (30.6)	68 (29.2)	3.04
High school (15~18 years old)	723 (71.6)	143 (69.4)	252 (75)	163 (69.4)	165 (70.8)	
**Residential regions** ^6^						
Capital region	428 (42.3)	83 (40.3)	161 (47.9)	89 (37.9)	95 (40.7)	28.441 *
Gangwon region	27 (2.7)	5 (2.4)	10 (3)	6 (2.6)	6 (2.6)	
Chungcheong region	143 (14.2)	33 (16)	49 (14.6)	41 (17.4)	20 (8.6)	
Jeolla region	119 (11.8)	28 (13.6)	31 (9.2)	23 (9.8)	37 (15.9)	
Gyeongsang region	278 (27.5)	56 (27.2)	77 (22.9)	71 (30.2)	74 (31.8)	
Jeju region	15 (1.5)	1 (0.5)	8 (2.5)	5 (2.1)	1 (0.4)	
**Research site**						
City area	750 (74.3)	160 (77.7)	256 (76.2)	161 (68.5)	173 (74.2)	5.97
Rural area ^7^	260 (25.7)	46 (22.3)	80 (23.8)	74 (31.5)	60 (25.8)	
**Household type**						
With family	975 (96.5)	195 (94.7)	328 (97.6)	225 (95.7)	227 (97.4)	4.34
Alone	35 (3.5)	11 (5.3)	8 (2.4)	10 (4.3)	6 (2.6)	
**Economic status** ^8^						
Upper	77 (7.6)	20 (9.7)	14 (4.2)	24 (10.2)	19 (8.2)	36.02 ***
Upper-middle	259 (25.6)	66 (32)	82 (24.4)	70 (29.8)	41 (17.6)	
Middle	495 (49)	90 (43.7)	180 (53.6)	111 (47.2)	114 (48.9)	
Lower-middle	151 (15)	24 (11.7)	52 (15.4)	27 (11.5)	48 (20.6)	
Lower	28 (2.8)	6 (2.9)	8 (2.4)	3 (1.3)	11 (4.7)	

^1^ The four clusters have been identified using a cluster analysis of the DGA 24-item scores, and they have been assigned descriptive names that characterize the dietary adherence patterns of each cluster: Cluster 1 (selective intake-low support), Cluster 2 (regular habits-unbalanced intake), Cluster 3 (high adherence), Cluster 4 (low adherence); ^2^ *p*-values are from F and χ^2^ tests on cluster differences (* *p* < 0.05, *** *p* < 0.001); ^3^ Weight and height are self-reported; ^4^ Weight status is categorized according to body mass index (BMI) percentiles: a BMI under the 5th percentile indicates underweight, between the 5th and 85th percentiles indicates normal, the 85th to 95th percentiles indicates overweight, and at or above the 95th percentile indicates obesity; ^5^ Values within a row with identical letters were significantly different, as determined by analysis of variance with Tukey post hoc tests; ^6^ Regions were reclassified as follows: Capital region (Seoul, Gyeonggi-do, Incheon), Gangwon region (Gangwon-do), Chungcheong region (Daejeon, Sejong, Chungcheongbuk-do, Chungcheongnam-do), Jeolla region (Gwangju, Jeollabuk-do, Jeollanam-do), Gyeongsang region (Busan, Daegu, Ulsan, Gyeongsangbuk-do, Gyeongsangnam-do), and Jeju region (Jeju Special Self-Governing Province); ^7^ The administrative divisions of the Republic of Korea include eup (towns), myeon (townships), and ri (villages) as rural units under provincial and city jurisdictions; ^8^ Economic status is self-reported.

**Table 3:** Distribution of “Excellent,” “Fair”, and “Poor” dietary quality ratings by cluster ^1^ membership.

Component Grade	Total (N = 1010)	Cluster 1 (N = 206, 20.4%)	Cluster 2 (N = 336, 33.3%)	Cluster 3 (N = 235, 23.2%)	Cluster 4 (N = 233, 23.1%)	χ^2 2^
	Sample size (weighted %)					
**Total DGA**						
Excellent ^3^	256 (25.3)	24 (11.7)	19 (5.7)	213 (90.6)	0 (0.0)	1222.2 ***
Fair	503 (49.8)	154 (74.7)	292 (86.9)	22 (9.4)	35 (15)	
Poor	251 (24.9)	28 (13.6)	25 (7.4)	0 (0.0)	198 (85)	
**Food intake**						
Excellent	252 (25)	67 (32.5)	8 (2.4)	175 (74.5)	2 (0.9)	603.4 ***
Fair	508 (50.3)	122 (59.2)	230 (68.5)	60 (25.5)	96 (41.2)	
Poor	250 (24.8)	17 (8.3)	98 (29.2)	0 (0.0)	135 (57.9)	
**Dietary and physical activity behaviors**						
Excellent	247 (24.5)	34 (16.5)	71 (21.1)	138 (58.7)	4 (1.7)	347.46 ***
Fair	510 (50.5)	122 (59.2)	205 (61)	90 (38.3)	93 (39.9)	
Poor	253 (25)	50 (24.3)	60 (17.9)	7 (3)	136 (58.4)	
**Dietary culture**						
Excellent	256 (25.3)	20 (9.7)	95 (28.3)	141 (60)	0 (0.0)	636.99 ***
Fair	503 (49.8)	127 (61.7)	229 (68.2)	94 (40)	53 (22.7)	
Poor	251 (24.9)	59 (28.6)	12 (3.6)	0 (0.0)	180 (77.3)	

^1^ The four clusters have been identified using a cluster analysis of the DGA 24-item scores, and they have been assigned descriptive names that characterize the dietary adherence patterns of each cluster: Cluster 1 (selective intake-low support), Cluster 2 (regular habits-unbalanced intake), Cluster 3 (high adherence), Cluster 4 (low adherence); ^2^ *p*-values are from χ^2^ tests on cluster differences (*** *p* < 0.001); ^3^ Scores are presented as mean ± SD. Evaluation criteria were classified into three levels: Excellent (75–100%), Fair (25–74.9%), and Poor (0–24.9%). For the total DGA score, Excellent = 62.6–100, Fair = 45.9–62.5, and Poor = 0–45.8. For food intake, Excellent = 48.1–100, Fair = 28.8–48.0, and Poor = 0–28.7. For dietary and physical activity behaviors, Excellent = 63.0–100, Fair = 40.5–62.9, and Poor = 0–40.4. For dietary culture, Excellent = 78.0–100, Fair = 56.5–77.9, and Poor = 0–56.4.

**Table 4:** Distribution of food literacy scores and grades by cluster ^1^ membership.

Food Literacy (FL)	Total (N = 1010)	Cluster 1 (N = 206, 20.4%)	Cluster 2 (N = 336, 33.3%)	Cluster 3 (N = 235, 23.2%)	Cluster 4 (N = 233, 23.1%)	F/χ^2 2^
	Mean ± SD					
Total FL	52.78 ±16.40	52.72 ± 14.56 ^b3^	50.96 ± 14.39 ^b^	64.06 ± 15.68 ^c^	44.05 ± 15.02 ^a^	73.42 ***
Selection	61.83 ± 18.68	59.55 ± 17.29 ^b^	63.27 ± 16.77 ^c^	72.47 ± 16.36 ^d^	51.03 ± 18.37 ^a^	62.88 ***
Production	36.36 ± 24.45	35.26 ± 23.40 ^b^	33.7 ± 23.03 ^b^	48.30 ± 26.57 ^c^	29.12 ± 20.73 ^a^	29.20 ***
Distribution	48.18 ± 25.24	48.41 ± 24.58 ^b^	45.37 ± 23.43 ^b^	61.71 ± 24.69 ^c^	38.40 ± 23.15 ^a^	39.67 ***
Preparation and cooking	63.62 ± 20.86	63.81 ± 19.47 ^b^	62.58 ± 20.12 ^b^	71.34 ± 19.15 ^c^	57.16 ± 22.36 ^a^	19.46 ***
Intake	54.33 ± 20.57	56.87 ± 18.27 ^c^	50.58 ± 18.55 ^b^	66.01 ± 18.62 ^d^	45.74 ± 21.44 ^a^	49.92 ***
**Component grade ^4^**	Sample size (%)					
Upper	283 (28.0)	55 (26.7)	72 (21.4)	133 (56.6)	23 (9.9)	181.63 ***
Upper middle	290 (28.7)	65 (31.6)	114 (33.9)	58 (24.7)	53 (22.7)	
Lower middle	218 (21.6)	50 (24.3)	72 (21.4)	28 (11.9)	68 (29.2)	
Lower	219 (21.7)	36 (17.5)	78 (23.2)	16 (6.8)	89 (38.2)	

^1^ The four clusters have been identified using a cluster analysis of the Dietary Guideline Adherence (DGA) 24-item scores, and they have been assigned descriptive names that characterize the dietary adherence patterns of each cluster: Cluster 1 (selective intake-low support), Cluster 2 (regular habits-unbalanced intake), Cluster 3 (high adherence), Cluster 4 (low adherence); ^2^ *p*-values are from F and χ^2^ tests on cluster differences (*** *p* < 0.001); ^3^ Values within a row with identical letters were significantly different (*p* < 0.05), as determined by analysis of variance with Tukey post hoc tests; ^4^ Evaluation criteria were classified into four levels from total FL score: Upper = 100–62, Upper middle = 51–61, Lower middle = 41–50, Lower = 0–40.

**Table 5:** Correlation analysis of each DGA and FL domain.

	Total DGA	FI	DPB	DC	Total FL	Selection	Production	Distribution	Preparation and Cooking	Intake
Total DGA ^1^	1									
FI ^2^	0.773 **^6^	1								
DPB ^3^	0.768 **	0.404 **	1							
DC ^4^	0.793 **	0.412 **	0.413 **	1						
Total FL ^5^	0.496 **	0.407 **	0.334 **	0.402 **	1					
Selection	0.437 **	0.290 **	0.298 **	0.423 **	0.789 **	1				
Production	0.330 **	0.301 **	0.213 **	0.239 **	0.705 **	0.364 **	1			
Distribution	0.367 **	0.315 **	0.240 **	0.291 **	0.833 **	0.364 **	0.537 **	1		
Preparation and cooking	0.276 **	0.202 **	0.177 **	0.257 **	0.603 **	0.611 **	0.281 **	0.281 **	1	
Intake	0.429 **	0.384 **	0.312 **	0.297 **	0.725 **	0.456 **	0.446 **	0.426 **	0.406 **	1

^1^ DGA, Dietary guideline adherence; ^2^ FI, Food intake; ^3^ DPB, Dietary and physical activity behaviors; ^4^ DC, Dietary culture; ^5^ FL, Food literacy; ^6^ *p*-value was determined by correlation analysis (** *p* < 0.01).

**Table 6:** Linear regression results for the association between food literacy and DGA score.

Variable	B	SE ^2^	β	t	*p*-Value ^3^
(constant)	16.157	2.07			
Total DGA ^1^	0.672	0.037	0.496	18.13	<0.001
F = 328.7; *p* < 0.001; R^2^ = 0.246; adj.R^2^ = 0.245					

^1^ DGA, Dietary Guideline Adherence; ^2^ SE, standard error; ^3^ *p*-value was determined by linear multiple regression analysis.

## Data Availability

The data presented in this study are available on request from the corresponding author due to privacy and ethical restrictions related to the protection of participants’ personal data.

## References

[1] van Sluijs E.M.F., Ekelund U., Crochemore-Silva I., Guthold R., Ha A., Lubans D., Oyeyemi A.L., Ding D., Katzmarzyk P.T. (2021). Physical activity behaviours in adolescence: Current evidence and opportunities for intervention. Lancet.

[2] Chaudhary A., Sudzina F., Mikkelsen B.E. (2020). Promoting healthy eating among young people—A review of the evidence of the impact of school-based interventions. Nutrients.

[3] Arafa A., Yasui Y., Kokubo Y., Kato Y., Matsumoto C., Teramoto M., Nosaka S. (2024). Lifestyle behaviors of childhood and adolescence: Contributing factors, health consequences, and potential interventions. Am. J. Lifestyle Med..

[4] Centers for Disease Control and Prevention (2013). Trends in the prevalence of excess dietary sodium intake—United States, 2003–2010. Morb. Mortal. Wkly. Rep..

[5] Snetselaar L.G., de Jesus J.M., DeSilva D.M., Stoody E.E. (2021). Dietary Guidelines for Americans, 2020–2025. Nutr. Today.

[6] U.S. Department of Agriculture, U.S. Department of Health and Human Services (2020). Dietary Guidelines for Americans, 2020–2025. https://www.dietaryguidelines.gov/sites/default/files/2021-03/Dietary_Guidelines_for_Americans-2020-2025.pdf.

[7] Moore L.V., Thompson F.E., Demissie Z. (2017). Percentage of youth meeting federal fruit and vegetable intake recommendations, Youth Risk Behavior Surveillance System, United States and 33 states, 2013. J. Acad. Nutr. Diet.

[8] Bowman S.A., Clemens J.C., Friday J.E., Thoerig R.C., Moshfegh A.J. (2019). Added Sugars in American Children’s Diet: What We Eat in America, NHANES 2015–2016 (Food Surveys Research Group Data Brief). U.S. Department of Agriculture, Agricultural Research Service. https://www.ars.usda.gov/ARSUserFiles/80400530/pdf/DBrief/26_Sources%20of%20Added%20Sugars%20in%20Children’s%20Diet_1516.pdf.

[9] Food and Agriculture Organization of the United Nations (2025). Food-Based Dietary Guidelines—Republic of Korea. http://www.fao.org/nutrition/education/food-dietary-guidelines/regions/countries/republic-of-korea/en/.

[10] Yun B., Kye S. (2024). Analysis of socio-demographic and dietary factors associated with fruit and vegetable consumption among Korean adolescents: Use of data from the 7th and 8th Korea National Health and Nutrition Examination Survey (2016–2019). J. Nutr. Health.

[11] Hwang S.B., Park S., Jin G.R., Jung J.H., Park H.J., Lee S.H., Shin S., Lee B.H. (2020). Trends in Beverage Consumption and Related Demographic Factors and Obesity among Korean Children and Adolescents. Nutrients.

[12] Kang M., Choi S.Y., Jung M. (2021). Dietary intake and nutritional status of Korean children and adolescents: A review of national survey data. Clin. Exp. Pediatr..

[13] Lee B.R., Ryu H.K. (2023). Trends in obesity prevalence among Korean adolescents and analysis of factors related to obesity. J. Nutr. Educ. Behav..

[14] Willett W., Rockström J., Loken B., Springmann M., Lang T., Vermeulen S., Garnett T., Tilman D., DeClerck F., Wood A. (2019). Food in the Anthropocene: The EAT–Lancet Commission on healthy diets from sustainable food systems. Lancet.

[15] Richonnet C., Mosser F., Favre E., Robert M., Martin F., Thiebaut I. (2021). Nutritional quality and degree of processing of children’s foods assessment on the French market. Nutrients.

[16] Uddin R., Lee E.Y., Khan S.R., Tremblay M.S., Khan A. (2019). Clustering of lifestyle risk factors for non-communicable diseases in 304,779 adolescents from 89 countries: A global perspective. Prev. Med..

[17] Veronese N., Notarnicola M., Cisternino A.M., Inguaggiato R., Guerra V., Reddavide R., Donghia R., Rotolo O., Zinzi I., Leandro G. (2020). Trends in adherence to the Mediterranean diet in South Italy: A cross sectional study. Nutr. Metab. Cardiovasc. Dis..

[18] Sinai T., Axelrod R., Shimony T., Boaz M., Kaufman-Shriqui V. (2021). Dietary Patterns among Adolescents Are Associated with Growth, Socioeconomic Features, and Health-Related Behaviors. Foods.

[19] Liu K.S.N., Chen J.Y., Ng M.Y.C., Yeung M.H.Y., Bedford L.E., Lam C.L.K. (2021). How does the family influence adolescent eating habits in terms of knowledge, attitudes and practices? A global systematic review of qualitative studies. Nutrients.

[20] van Ansem W.J., Schrijvers C.T., Rodenburg G., van de Mheen D. (2014). Maternal educational level and children’s healthy eating behaviour: Role of the home food environment (Cross-sectional results from the INPACT Study). Int. J. Behav. Nutr. Phys. Act..

[21] Lim J., Lee S., Hwang J.Y., Oh J. (2025). Empowering healthy adolescents: A dietary adherence tool incorporating environmental factors based on Korean guidelines. Nutrients.

[22] Cullen T., Hatch J., Martin W., Higgins J.W., Sheppard R. (2015). Food literacy: Definition and framework for action. Can. J. Diet. Pract. Res..

[23] Patton G.C., Sawyer S.M., Santelli J.S., Ross D.A., Afifi R., Allen N.B., Arora M., Azzopardi P., Baldwin W., Bonell C. (2016). Our future: A Lancet Commission on adolescent health and wellbeing. Lancet.

[24] Mura Paroche M., Caton S.J., Vereijken C.M.J.L., Weenen H., Houston-Price C. (2017). How infants and young children learn about food: A systematic review. Front. Psychol..

[25] Utter J., Larson N., Laska M.N., Winkler M., Neumark-Sztainer D. (2018). Self-perceived cooking skills in emerging adulthood predict better dietary behaviors and intake 10 years later: A longitudinal study. J. Nutr. Educ. Behav..

[26] Bailey C.J., Drummond M.J., Ward P.R. (2019). Food literacy programmes in secondary schools: A systematic literature review and narrative synthesis of quantitative and qualitative evidence. Public Health Nutr..

[27] Park D., Choi M.K., Park Y.K., Park C.Y., Shin M.J. (2022). Higher food literacy scores are associated with healthier diet quality in children and adolescents: The development and validation of a two-dimensional food literacy measurement tool for children and adolescents. Nutr. Res. Pract..

[28] Devlin U.M., McNulty B.A., Nugent A.P., Gibney M.J. (2012). The use of cluster analysis to derive dietary patterns: Methodological considerations, reproducibility, validity and the effect of energy mis-reporting. Proc. Nutr. Soc..

[29] Hearty A.P., Gibney M.J. (2009). Comparison of cluster and principal component analysis techniques to derive dietary patterns in Irish adults. Br. J. Nutr..

[30] Milligan G.W., Cooper M.C. (1987). Methodology review: Clustering methods. Appl. Psychol. Meas..

[31] Hennig C., Meila M., Murtagh F., Rocci R. (2015). Handbook of Cluster Analysis.

[32] Crozier S.R., Robinson S.M., Borland S.E., Inskip H.M. (2006). Dietary patterns in the Southampton Women’s Survey. Eur. J. Clin. Nutr..

[33] Fielding-Singh P., Fan J.X. (2024). Dietary patterns among US children: A cluster analysis. J. Acad. Nutr. Diet..

[34] Bailey R.L., Gutschall M.D., Mitchell D.C. (2006). Comparative strategies for using cluster analysis to assess dietary patterns. J. Am. Diet. Assoc..

[35] Liese A.D., Krebs-Smith S.M., Subar A.F., George S.M., Harmon B.E., Neuhouser M.L., Boushey C.J., Schap T.E., Reedy J. (2015). The Dietary Patterns Methods Project: Synthesis of findings across cohorts and relevance to dietary guidance. J. Nutr..

[36] Bonaccorso G. (2019). Hands-On Unsupervised Learning with Python: Implement Machine Learning and Deep Learning Models Using Scikit-Learn, TensorFlow, and More.

[37] Milligan G.W., Cooper M.C. (1985). An examination of procedures for determining the number of clusters in a data set. Psychometrika.

[38] Hennig C. (2015). What are the true clusters?. Pattern Recognit. Lett..

[39] Stricker M.D., Onland-Moret N.C., Boer J.M.A., van der Schouw Y.T., Verschuren W.M.M., May A.M., Beulens J.W.J. (2013). Dietary patterns derived from principal component- and k-means cluster analysis: Long-term association with coronary heart disease and stroke. Nutr. Metab. Cardiovasc. Dis..

[40] Geraets A.F.J., Heinz A. (2023). The associations of dietary habits with health, well-being, and behavior in adolescents: A cluster analysis. Child Care Health Dev..

[41] Villegas R., Salim A., Collins M.M., Flynn A., Perry I.J. (2004). Dietary patterns in middle-aged Irish men and women defined by cluster analysis. Public Health Nutr..

[42] So H., Park D., Choi M.K., Kim Y.S., Shin M.J., Park Y.K. (2021). Development and validation of a food literacy assessment tool for community-dwelling elderly people. Int. J. Environ. Res. Public Health.

[43] Gwon D., Hwang J.Y., Oh J. (2025). Nutrition quotient for preschoolers and key impacting factors in Korea: A cross-sectional study on food literacy, social support, and the food environment of primary caregivers. Korean J. Community Nutr..

[44] Greenwood D., Cade J., Draper A., Barrett J., Calvert C., Greenhalgh A. (2000). Seven unique food consumption patterns identified among women in the UK Women’s Cohort Study. Eur. J. Clin. Nutr..

[45] Giampieri F., Rosi A., Scazzina F., Frias-Toral E., Abdelkarim O., Aly M., Zambrano-Villacres R., Pons J., Vázquez-Araújo L., Sumalla Cano S. (2024). Youth Healthy Eating Index (YHEI) and diet adequacy in relation to country-specific national dietary recommendations in children and adolescents in five Mediterranean countries from the DELICIOUS project. Nutrients.

[46] Moraeus L., Lindroos A.K., Warensjö Lemming E., Mattisson I. (2020). Diet diversity score and healthy eating index in relation to diet quality and socio-demographic factors: Results from a cross-sectional national dietary survey of Swedish adolescents. Public Health Nutr..

[47] Gu X., Tucker K.L. (2017). Dietary quality of the US child and adolescent population: Trends from 1999 to 2012 and associations with the use of federal nutrition assistance programs. Am. J. Clin. Nutr.

[48] Thomson J.L., Tussing-Humphreys L.M., Goodman M.H., Landry A.S. (2019). Diet quality in a nationally representative sample of American children by sociodemographic characteristics. Am. J. Clin. Nutr..

[49] Vandevijvere S., Geelen A., Gonzalez-Gross M., van’t Veer P., Dallongeville J., Mouratidou T., Dekkers A., Börnhorst C., Breidenassel C., Crispim S.P. (2013). Evaluation of food and nutrient intake assessment using concentration biomarkers in European adolescents from the Healthy Lifestyle in Europe by Nutrition in Adolescence study. Br. J. Nutr..

[50] Hanley-Cook G.T., Hoogerwerf S., Parraguez J.P., Gie S.M., Holmes B.A. (2024). Minimum dietary diversity for adolescents: Multicountry analysis to define food group thresholds predicting micronutrient adequacy among girls and boys aged 10–19 years. Curr. Dev. Nutr..

[51] Monge-Rojas R., Vargas-Quesada R., Gómez G. (2022). Role of residence area on diet diversity and micronutrient intake adequacy in urban and rural Costa Rican adolescents. Nutrients.

[52] Braune T., Adams J., Winpenny E.M. (2024). Exploring the changing association between parental and adolescent fruit and vegetable intakes, from age 10 to 30 years. Int. J. Behav. Nutr. Phys. Act..

[53] Parks C.A., Blaser C., Smith T.M., Calloway E.E., Oh A.Y., Dwyer L.A., Yaroch A.L. (2018). Correlates of fruit and vegetable intake among parents and adolescents: Findings from the Family Life, Activity, Sun, Health, and Eating (FLASHE) study. Public Health Nutr..

[54] Reicks M., Banna J., Cluskey M., Gunther C., Hongu N., Richards R., Topham G., Wong S.S. (2015). Influence of parenting practices on eating behaviors of early adolescents during independent eating occasions: Implications for obesity prevention. Nutrients.

[55] Pearson N., Griffiths P., Biddle S.J., Johnston J.P., Haycraft E. (2017). Individual, behavioural and home environmental factors associated with eating behaviours in young adolescents. Appetite.

[56] Yim H.R., Yun H.J., Lee J.H. (2021). An investigation on Korean adolescents’ dietary consumption: Focused on sociodemographic characteristics, physical health, and mental health. Int. J. Environ. Res. Public Health.

[57] Müllertz A.L.O., Stjernqvist N.W., Outzen M.H., Bloch P., Elsborg P., Ravn-Haren G. (2024). A cross-sectional study of the association between food literacy and dietary intake among Danish adolescents. Appetite.

[58] Otsuka Y., Kaneita Y., Itani O., Jike M., Osaki Y., Higuchi S., Kanda H. (2020). Gender differences in dietary behaviors among Japanese adolescents. Prev. Med. Rep..

[59] Fayet F.M., McConnell A., Tuck K., Petocz P. (2017). Breakfast and breakfast cereal choice and its impact on nutrient and sugar intakes and anthropometric measures among a nationally representative sample of Australian children and adolescents. Nutrients.

[60] Ramsay S.A., Bloch T.D., Marriage B., Shriver L.H., Spees C.K., Taylor C.A. (2018). Skipping breakfast is associated with lower diet quality in young US children. Eur. J. Clin. Nutr..

[61] Askari M., Heshmati J., Shahinfar H., Tripathi N., Daneshzad E. (2020). Ultra-processed food and the risk of overweight and obesity: A systematic review and meta-analysis of observational studies. Int. J. Obes..

[62] Rodrigues P.R.M., Luiz R.R., Monteiro L.S., Ferreira M.G., Goncalves-Silva R.M.V., Pereira R.A. (2017). Adolescents’ unhealthy eating habits are associated with meal skipping. Nutrition.

[63] Sila S., Ilić A., Mišigoj-Duraković M., Sorić M., Radman I., Šatalić Z. (2019). Obesity in adolescents who skip breakfast is not associated with physical activity. Nutrients.

[64] Lehikoinen E., Salonen A.O. (2019). Food preferences in Finland: Sustainable diets and their differences between groups. Sustainability.

[65] Isenhour C., Ardenfors M. (2009). Gender and sustainable consumption: Policy implications. Int. J. Innov. Sustain. Dev..

[66] Lee Y.J., Kim T.H. (2022). A study on components of food literacy competencies: Focused on the differences in perception by generation and food-educational experience. Culi Sci. Hos Res..

[67] Jo J.I., Kim H.K. (2008). Food habits and eating snack behaviors of middle school students in Ulsan area. Korean J. Nutr..

[68] Berge J.M., MacLehose R.F., Larson N., Laska M., Neumark-Sztainer D. (2016). Family food preparation and its effects on adolescent dietary quality and eating patterns. J. Adolesc. Health.

[69] Dave J.M., Evans A.E., Condrasky M.D., Williams J.E. (2012). Parent-reported social support for child’s fruit and vegetable intake: Validity of measures. J. Nutr. Educ. Behav..

[70] Hearn M.D., Baranowski T., Baranowski J., Doyle C., Smith M., Lin L.S., Resnicow K. (1998). Environmental influences on dietary behavior among children: Availability and accessibility of fruits and vegetables enable consumption. J. Health Educ..

[71] Pyper E., Harrington D., Manson H. (2016). The impact of different types of parental support behaviours on child physical activity, healthy eating, and screen time: A cross-sectional study. BMC Public Health.

[72] Bae E.J., Yoon J.Y. (2023). Unhealthy weight control behaviors and related factors by gender and weight status: Results from a nationally representative sample of Korean adolescents. Arch. Psychiatr. Nurs..

[73] Kim Y., Austin S.B., Subramanian S.V., Thomas J.J., Eddy K.T., Franko D.L., Rodgers R.F., Kawachi I. (2018). Risk factors for disordered weight control behaviors among Korean adolescents: Multilevel analysis of the Korea Youth Risk Behavior Survey. Int. J. Eat. Disord..

[74] Kidwell K.M., Milligan M.A., Deyo A., Lasker J., Vrabec A. (2024). Emotional eating prevalence and correlates in adolescents in the United States. Child. Obes..

[75] Weng H., Barnhart W.R., Zickgraf H.F., Dixit U., Cheng Y., Chen G., He J. (2023). Negative emotional eating patterns in Chinese adolescents: A replication and longitudinal extension with latent profile and transition analyses. Appetite.

[76] Joseph P.L., Gonçalves C., Fleary S.A. (2023). Psychosocial correlates in patterns of adolescent emotional eating and dietary consumption. PLoS ONE.

[77] Ramírez-Garza S.L., Laveriano-Santos E.P., Moreno J.J., Bodega P., de Cos-Gandoy A., de Miguel M., Santos-Beneit G., Fernández-Alvira J.M., Fernández-Jiménez R., Martínez-Gómez J. (2023). Metabolic syndrome, adiposity, diet, and emotional eating are associated with oxidative stress in adolescents. Front. Nutr..

[78] Shatwan I.M., Alzharani M.A. (2024). Association between perceived stress, emotional eating, and adherence to healthy eating patterns among Saudi college students: A cross-sectional study. J. Health Popul. Nutr..

[79] Ronto R., Ball L., Pendergast D., Harris N. (2016). Adolescents’ perspectives on food literacy and its impact on their dietary behaviours. Appetite.

[80] Ghorbany S., Hu M., Yao S., Wang C., Sisk M., Nguyen Q.C., Zhang K. (2025). Intersecting paths to health: A factor analysis approach to socioeconomic and environmental determinants in Indiana. Int. J. Environ. Res. Public Health.

[81] Han J.S., Kim S.S., Go M.A. (2015). Influences of dietary education activity on education satisfaction and self-respect. Culi. Sci. Hos. Res..

[82] Rousham O., Braune T., Mori T.A., Beilin L.J., Winpenny E.M. (2025). Parental and individual socioeconomic position show distinct associations with trajectories of diet quality across adolescence and early adulthood. Appetite.

[83] Hinnig P.D.F., Monteiro J.S., De Assis M.A.A., Levy R.B., Peres M.A., Perazi F.M., Porporatti A.L., Canto G.D.L. (2018). Dietary patterns of children and adolescents from high, medium and low human development countries and associated socioeconomic factors: A systematic review. Nutrients.

[84] Kurotani K., Shinsugi C., Takimoto H. (2021). Diet quality and household income level among students: 2014 national health and nutrition survey Japan. Eur. J. Clin. Nutr..

[85] Li Z., Xie S., Chen W. (2025). The influence of environment on adolescents’ physical exercise behavior based on family community and school micro-systems. Sci. Rep..

[86] Pluta B., Korcz A., Krzysztoszek J., Bronikowski M., Fumagalli G., Morina B. (2020). Associations between adolescents’ physical activity behavior and their perceptions of parental, peer and teacher support. Arch. Public Health.

[87] Chung A., Vieira D., Donley T., Tan N., Jean-Louis G., Kiely Gouley K., Seixas A. (2021). Adolescent peer influence on eating behaviors via social media: Scoping review. J. Med. Internet Res..

[88] Chong M.F.F. (2022). Dietary trajectories through the life course: Opportunities and challenges. Br. J. Nutr..

[89] Winpenny E.M., Penney T.L., Corder K., White M., Van Sluijs E.M. (2017). Change in diet in the period from adolescence to early adulthood: A systematic scoping review of longitudinal studies. Int. J. Behav. Nutr. Phys. Act..

[90] Hunter E., Stone R.A., Brown A., Hardman C.A., Johnstone A.M., Greatwood H.C., Dineva M., Douglas F. (2025). “We go hunting …”: Understanding experiences of people living with obesity and food insecurity when shopping for food in the supermarket to meet their weight-related goals. Appetite.

[91] Puddephatt J.A., Keenan G.S., Fielden A., Reaves D.L., Halford J.C.G., Hardman C.A. (2020). ‘Eating to survive’: A qualitative analysis of factors influencing food choice and eating behaviour in a food-insecure population. Appetite.

[92] Robinson E., Jones A., Marty L. (2022). The role of health-based food choice motives in explaining the relationship between lower socioeconomic position and higher BMI in UK and US adults. Int. J. Obes..

[93] The Food Foundation (2025). The Broken Plate 2025. https://foodfoundation.org.uk/publication/broken-plate-2025.

[94] Stone R.A., Christiansen P., Johnstone A.M., Brown A., Douglas F., Hardman C.A. (2025). Understanding the barriers to purchasing healthier, more environmentally sustainable food for people living with obesity and varying experiences of food insecurity in the UK. Food Policy.

[95] Drewnowski A. (2022). Food insecurity has economic root causes. Nat. Food.

[96] Li H., Choi J., Kim A., Liu G. (2024). Association between physical activity, smartphone usage, and obesity risk among Korean adolescents: A cross-sectional study based on 2021 Korean adolescent health behavior survey. Acta Psychol.

[97] Sulz L.D., Gleddie D.L., Kinsella C., Humbert M.L. (2023). The health and educational impact of removing financial constraints for school sport. Eur. Phys. Educ. Rev..

[98] Ghazarian S.R., Roche K.M. (2010). Social support and low-income, urban mothers: Longitudinal associations with adolescent delinquency. J. Youth Adolesc..

[99] Quader Z.S., Gillespie C., Sliwa S.A., Ahuja J.K., Burdg J.P., Moshfegh A., Pehrsson P.R., Gunn J.P., Mugavero K., Cogswell M.E. (2017). Sodium intake among US school-aged children: National Health and Nutrition Examination Survey, 2011–2012. J. Acad. Nutr. Diet.

[100] Park S.K., Lee J.H. (2020). Factors influencing the consumption of convenience foods among Korean adolescents. J. Nutr. Health.

